# Numerical Analysis of Space Deployable Structure Based on Shape Memory Polymers

**DOI:** 10.3390/mi12070833

**Published:** 2021-07-17

**Authors:** Zepeng He, Yang Shi, Xiangchao Feng, Zhen Li, Yan Zhang, Chunai Dai, Pengfei Wang, Liangyu Zhao

**Affiliations:** 1 Beijing Institute of Technology, School of Aerospace Engineering, Beijing 100081, China; 2 Qian Xuesen Laboratory of Space Technology, China Academy of Space Technology, Beijing 100094, China; 19126255@bjtu.edu.cn (Y.S.); fengxiangchao@qxslab.cn (X.F.); zhangyan@qxslab.cn (Y.Z.); 3 School of Science, Beijing Jiaotong University, Beijing 100044, China; chadai@bjtu.edu.en

**Keywords:** shape memory polymers, finite element method, time–temperature equivalence principle, shape memory characteristics, space structure

## Abstract

Shape memory polymers (SMPs) have been applied in aerospace engineering as deployable space structures. In this work, the coupled finite element method (FEM) was established based on the generalized Maxwell model and the time–temperature equivalence principle (TTEP). The thermodynamic behavior and shape memory effects of a single-arm deployment structure (F-DS) and four-arm deployment structure (F-DS) based on SMPs were analyzed using the coupled FEM. Good consistency was obtained between the experimental data and simulation data for the tensile and S-DS recovery forces, verifying that the coupled FEM can accurately and reliably describe the thermodynamic behavior and shape memory effects of the SMP structure. The step-by-step driving structure is suitable for use as a large-scale deployment structure in space. This coupled FEM provides a new direction for future research on epoxy SMPs.

## 1. Introduction

SMPs, as programmable phase change materials, can be deformed to a temporary shape under given conditions and then reversed to their original permanent shape upon external stimulus [1,2,3,4]. Compared with other traditional SMPs, such as shape memory alloys and shape memory ceramics, SMPs have the advantages of light weight, low cost, good biocompatibility, and great deformation recovery [5,6,7,8,9,10,11,12,13,14,15]. Taking into account these excellent characteristics of SMPs, many researchers have applied them in the fields of aerospace [16,17,18,19], biomedical research [20,21,22], intelligent textiles [23], and self-healing materials [24,25,26].

SMPs, due to their advantages described herein, can be developed into technology platforms that allow the tailored multifunctional design. In this way, defined movements of implants triggered either directly or indirectly [27,28]; tailored mechanical properties [29,30]; and capability for sterilization [31,32], biodegradability [33], biocompatibility [34,35], and controlled drug release [33,36,37] can be achieved [20]. Lendlein et al. introduced a group of degradable thermoplastic polymers that were able to change their shape when the loaded temperature was increased, whereby the shape memory capability of the specimens enabled bulky implants to be placed in the body through small incisions and allowed complex mechanical deformation processes to be performed automatically [33]. Yakacki et al. developed novel SMP networks with capability for free recovery at body temperature, which were suitable for specific applications in minimally invasive cardiovascular devices [36]. Neffe et al. developed a ureteral stent using SMPs that can be anchored in the ureter, which could have a significant impact in terms of controlled drug release [38].

SMPs can be temporarily fixed and readily formed because they are strong below the glass transition temperature and deformable above the glass transition temperature [4,39,40,41]. The aforementioned excellent performance means that SMPs have great application potential in aerospace, especially for large deployable antenna structures in aerospace satellites, including hinges, solar panels, deployable panels, suspenders, and reflector antennas [42,43]. Leng et al. designed and fabricated a deployable hinge based on SMPs and used it in a deployable driving structure for deployable space solar panels [44]. A smart deployable space antenna with a diameter of 10 m based on SMPs was studied by NASA’s Air Propulsion Laboratory, in which the antenna could be deployed in orbit and the shape of the antenna was further maintained after deployment [45].

There have been many theoretical studies on SMPs [46], which can be categorized into studies on rheological models [9,47,48,49] and phase transition models [41,50,51,52,53,54]; however, the above models might be difficult to popularize to a certain degree, as they are hindered by the complexity of the parameter determination process [55]. Tao et al. simulated the shape memory effects of deployable hinged shells of SMPs using the UMAT compiled by ABAQUS [56]. The variable stiffness of an integrated SMPC hinge at different temperatures was calculated and simulated by Liu’s group, in which good shape memory performance was obtained [57]. Liu et al. simulated the bending and recovery process of a cylindrical tube using finite element analysis, and the stress distribution of the cylindrical tube in the bent state was obtained [58]; hence, in order to maintain the stability and reliability of a spacecraft in space, it is necessary to fully characterize the mechanical properties of space deployment structures based on SMPs. It is particularly important to develop a complete and reliable method of simulation analysis.

In this paper, a coupled FEM was established based on the generalized Maxwell model and TTEP. The mechanical deformation of the SMPs was decomposed into the hyperelastic part (which was expressed using the neo-Hookean model) and viscoelastic part (which was expressed using the generalized Maxwell model and Williams–Landel–Ferry (WLF) equations), considering the large deformation behavior of the studied object. The TTEP was used to describe the response characteristics of SMPs at different temperatures. Based on the experimental data from the tension and relaxation tests of the E-SMP, the numerical model parameters were calculated using ABAQUS. The accuracy of the numerical analysis model was verified by calculating the same analysis model as in the tensile experiment. The thermodynamic behavior and shape memory effect of the S-DS and F-DS of the electrically driven epoxy resin SMPs prepared in the laboratory were studied and analyzed. The variations of the torque in the *X*-axis and bending angle of the S-DS in the response process were calculated. The relative internal energy and stress cloud map of the F-DS during simultaneous and step-by-step driving were obtained.

## 2. Mechanical Characterization of the E-SMP

SMPs possess viscoelastic properties typical of polymers and unique shape memory properties. The materials considered in this paper were electrothermal E-SMP materials based on epoxy resin. According to the synthesis method for epoxy resin outlined in [41,53,54], multi-walled carbon nanotubes and carbon fiber powder were introduced into the material to enhance the mechanical and electrical properties of the E-SMPs.

### 2.1. Dynamic Mechanical Analyzer (DMA)

**Figure 1:**
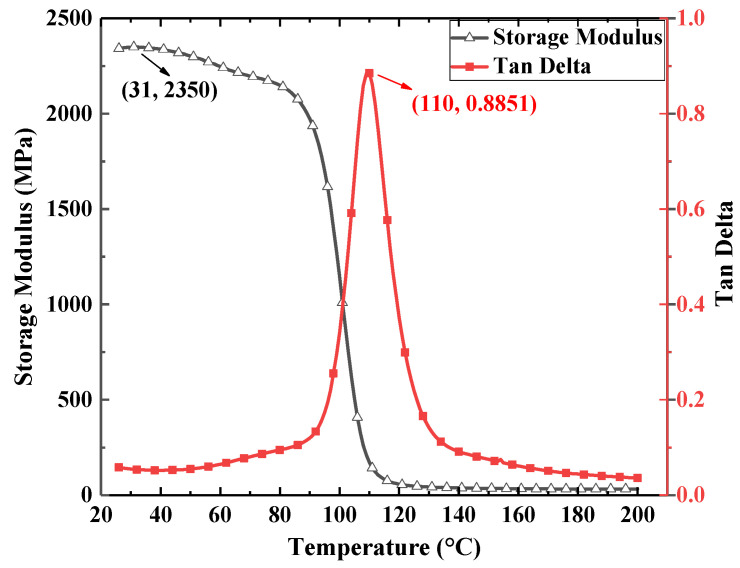
Dynamic thermomechanical analysis curve for the E-SMP material.

In order to obtain the high- and low-temperature moduli and glass transition temperature for E-SMP, the samples were tested by DMA. The sample size for E-SMP was 30 mm × 5 mm × 1 mm. According to the experimental references [59,60], the DMA measurements of the E-SMP material were obtained by increasing the temperature from 25 °C to 250 °C with a heat rate of 2 °C/min and applying sinusoidal alternating stress with an oscillation frequency of 1 Hz and a load of 0.01 N. Through experimental analysis, the maximum tan delta (phase angle) was obtained when the temperature reached up to 110 °C. It can be seen in Figure 1 that the E-SMPs had a glass transition temperature ($T_{g}$) of 110 °C, a high storage modulus of 2350 MPa (below $T_{g}$), and an electric driving effect.

### 2.2. Relaxation Test

**Figure 2:**
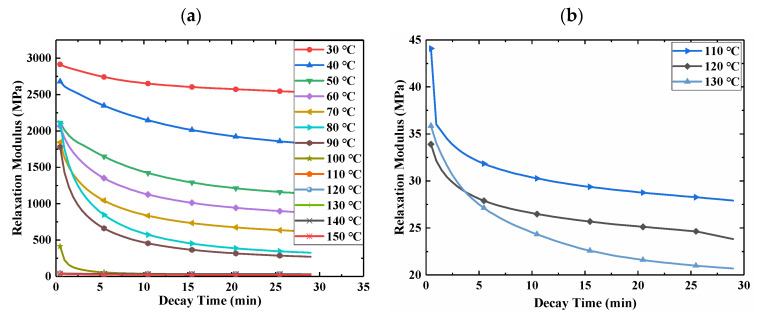
Relaxation test curves under different test temperatures: (**a**) relaxation test curves under different temperatures; (**b**) relaxation test curves under high temperatures.

In order to study the relaxation characteristics of E-SMP at different temperatures, the tensile testing machine with an environmental box was used to test the stress relaxation of the sample. The sample size for E-SMP was 30 mm × 5 mm × 1 mm. The test temperature range was from 30 °C to 150 °C, with temperature intervals of 10 °C. In the test, the loading rate was set to 2 mm/min, while the tensile elongation was 1 mm, which was kept for 1800 s. The relaxation data for E-SMP are shown in Figure 2.

In Figure 2a, it can be seen that under the given pre-strain conditions, the stress of the specimen at 30 °C was about 2971 MPa, which declined to 2534 MPa within 30 min. At lower temperatures, the material showed glassy properties and higher strength. When the temperature further exceeded 50 °C, E-SMP began to exhibit significant viscoelastic properties and the relaxation modulus decreased rapidly with the increase of the temperature. When heated to 110 °C, E-SMP changed to a rubbery state with high elastic strain, resulting in the modulus being basically unchanged. The test data were smoothed and filtered, as shown in Figure 2b. When the temperature was higher than the glass transition temperature of E-SMP, the material was in a rubber state. As derived from the curve, the modulus of E-SMP was very low at this time. The modulus of the material was between 20 and 45 MPa after 30 min attenuation.

### 2.3. Tensile Test

**Figure 3:**
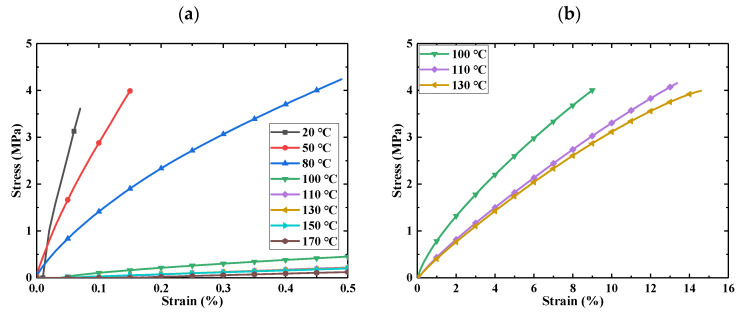
Tensile tests for E-SMP at different temperatures: (**a**) tensile characteristic curves of E-SMP at different temperatures; (**b**) tensile characteristic curves near the glass transition temperature.

Here, uniaxial tensile tests were carried out on the samples of E-SMP materials using a tensile testing machine with an environmental box. The test temperature includes 20, 50, 80, 100, 110, 130, 150, and 170 °C. The sample was stretched to the maximum measuring range of 18 N at a rate of 15 mm/min. The experimental data are shown in Figure 3.

It can be seen from the curves that the E-SMP material went through a glass state, viscoelastic state, and highly elastic rubber state. With the increases of temperature, the modulus of the E-SMP decreased gradually. When the temperature exceeded $T_{g}$, the E-SMP was in a highly elastic state and had almost no bearing capacity. Since the maximum measuring range of the instrument is only 18 N, the sample could not be broken.

### 2.4. Thermal Expansion Test

The linear thermal expansion coefficient was used to measure the thermal expansion coefficient of E-SMP. The equation is as follows:

(1) $$\alpha =\frac{1}{L}\times \frac{\Delta L}{\Delta T}$$

where L is the original length of the sample, ΔL is the rate of length change of the sample, and ΔT is the rate of temperature change.

**Figure 4:**
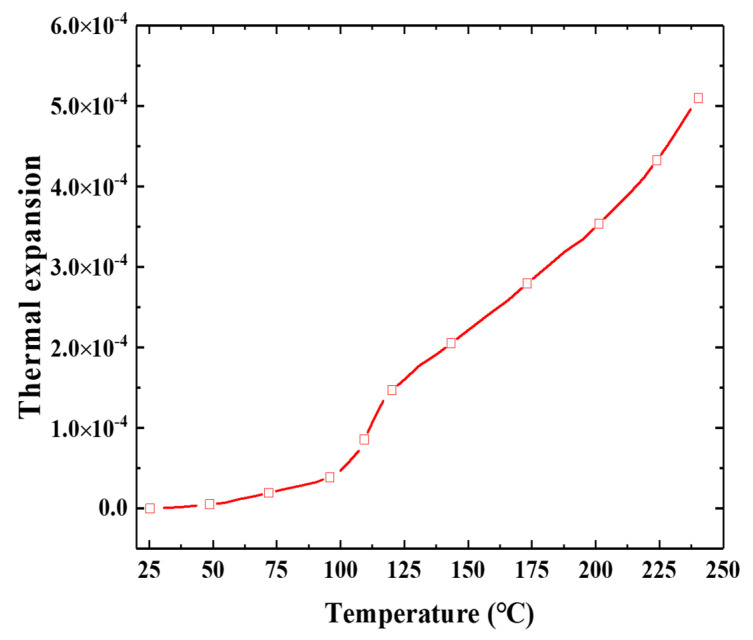
Thermal expansion coefficients of E-SMP at different temperatures.

The thermal expansion coefficients of E-SMP at different temperatures were measured using a thermomechanical test. The test temperature range was 25~250 °C, the heating rate was 1 °C/min, the pre-load was 2 N, and the material expanded freely. The experimental results are presented in Figure 4. It can be seen that the linear thermal expansion rate for E-SMP was relatively low at low temperatures (25~100 °C) because the material was glassy, while the linear thermal expansion rate increased sharply when the temperature increased to 100 °C owing to the E-SMP material transforming from a glassy state to a highly elastic state. The linear thermal expansion rate of the E-SMP material is high above the glass transition temperature (110 °C), at which temperature the material is in a rubber state.

## 3. Constitutive Modeling of the E-SMP

**Figure 5:**
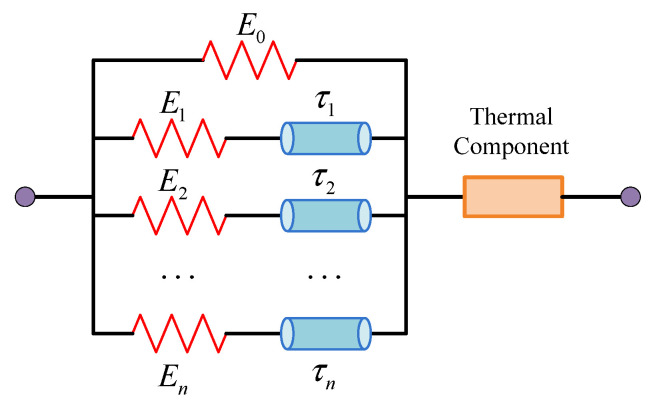
Generalized Maxwell model.

E-SMP has appealing shape memory effects. The constitutive equation used to characterize these effects is interesting. Referring to the deformation behavior of the material studied in this paper, the generalized Maxwell model and TTEP method outlined in [9,61] were used to describe the shape memory behavior of SMPs. The generalized Maxwell model consists of Maxwell and hyperelasticity components. It is assumed that the effect of the thermal expansion on SMPs is independent of the mechanical behavior, as shown in Figure 5, where $E_{0}$ is the Young’s modulus of the elastic term, $E_{i}$ is the Young’s modulus of the Maxwell element, $\tau_{i}$ presents the relaxation time of the Maxwell element, and n denotes the number of Maxwell components.

The strain Equation in the above model can be deduced as follows:

(2) $$\varepsilon_{Total}=\varepsilon_{M}+\varepsilon_{T}$$

where $\varepsilon_{Total}$ denotes the total strain of the model, $\varepsilon_{M}$ denotes the mechanical strain, and $\varepsilon_{T}$ denotes the thermal strain.

The thermal strain is defined as:

(3) $$\varepsilon_{T}=\alpha \left(T-T_{g}\right)$$

where α is the coefficient of thermal expansion, $T_{g}$ is the glass transition temperature, and T is the current temperature.

According to the theoretical model in [9], the constitutive model in the coupled FEM can be derived as:

(4) $$\sigma_{Total}\left(t\right)=E_{0}\varepsilon (t)+\varepsilon (t)\sum_{i=1}^{n}E_{i}e^{-t/\tau_{i}}$$

where $\sigma_{Total}\left(t\right)$ is the total stress; $E_{0}$ is the Young’s modulus of the elastic term; $E_{i}$ and $\tau_{i}$ are the Young’s modulus and relaxation time of the Maxwell element, respectively; ε(t) is the strain of the model; t is the real time; and n is the number of Maxwell components.

The mechanical deformation of E-SMP is decomposed into hyperelastic and viscoelastic parts; thus, the total strain energy can be expressed as the sum of the two parts [62]:

(5) $$W_{total}=W_{A}+W_{B}$$

where $W_{A}$ denotes the free energy of the rubbery state of the material and $W_{B}$ is the free energy of the viscoelastic part of the material.

For $W_{A}$, a hyperelastic term (instead of an elastic term) is added to enable a better agreement because of the large strain of the space expansion structure of SMPs [22]. Here, we chose the neo-Hookean hyperelastic equation:

(6) $$W_{A}=C_{10}\left({\bar{I}}_{1}-3\right)+\frac{1}{D_{1}}{\left(J_{el}-1\right)}^{2}$$

where ${\bar{I}}_{1}$ is the first stress invariant, $C_{10}$ and $D_{1}$ are the temperature correlation coefficients, and $J_{el}$ is the elastic volume strain, which can be obtained by fitting the experimental data.

Herein, the generalized Maxwell model and WLF equations were used to express $W_{B}$. According to the literature, the generalized Maxwell model and the WLF equation show the relationship between time and temperature. In addition, the model has already been shown to be reliable under a large deformation range [63]. In the generalized Maxwell model, the relaxation modulus G(t) can be expressed using the Prony series:

(7) $$G\left(t\right)=G_{\infty }+\sum_{i=1}^{n_{G}}G_{i}e^{-\tau /\tau_{i}^{G}}$$

where $G_{\infty }$ is the shear modulus in infinite time and $G_{i}$ is the shear modulus of the Maxwell element.

To obtain the effective relaxation modulus G(t), a Fourier transform is applied in Equation (7):

(8) $$G{\left(\omega \right)}^{2}=G_{s}{\left(\omega \right)}^{2}+G_{l}{\left(\omega \right)}^{2}$$

(9) $$G_{s}\left(\omega \right)=G_{0}+\sum_{i=1}^{n}\frac{G_{i}\tau_{i}^{2}\omega^{2}}{1+\tau_{i}^{2}\omega^{2}}$$

(10) $$G_{l}\left(\omega \right)=\sum_{i=1}^{n}\frac{G_{i}\tau_{i}\omega }{1+\tau_{i}^{2}\omega^{2}}$$

where $G_{s}\left(\omega \right)$ is the storage modulus, $G_{l}\left(\omega \right)$ is the loss modulus, $G_{0}$ is the initial shear modulus of the material, and $G_{i}$ and $\tau_{i}$ are a series of relaxation moduli and relaxation times, which can be obtained by fitting the force relaxation curve data at different temperatures.

To describe the response characteristics of SMPs at different temperatures, the TTEP was introduced [64]. It is known that prolonging the observation time and increasing the temperature are equivalent to the motion of molecules and the viscoelastic behavior of polymers; therefore, the WLF equation can be used to describe the time temperature effect of SMPs [65], which is shown as follows:

(11) $$\mathrm{lg}\left(a_{T}\right)=\frac{-C_{1}\left(T-T_{0}\right)}{C_{2}+T-T_{0}}$$

where $a_{T}\left(T\right)$ is the time temperature superposition shift factor in the WLF equation, $C_{1}$ and $C_{2}$ are material parameters, and $T_{0}$ is the reference temperature, which can be obtained by fitting the experimental data.

The relaxation time τ of the SMPs in the non-isothermal response process can be expressed by the following equation:

(12) $$\frac{d\tau }{dt}=\frac{1}{a_{T}\left(T\left(t\right)\right)}$$

The theoretical model parameters mentioned above can be processed using the material module in ABAQUS. By using ABAQUS [66,67], the analysis efficiency of the E-SMP structure can be greatly improved.

## 4. Material Parameters Calibration

### 4.1. Hyperelastic Parameters Calibration

**Figure 6:**
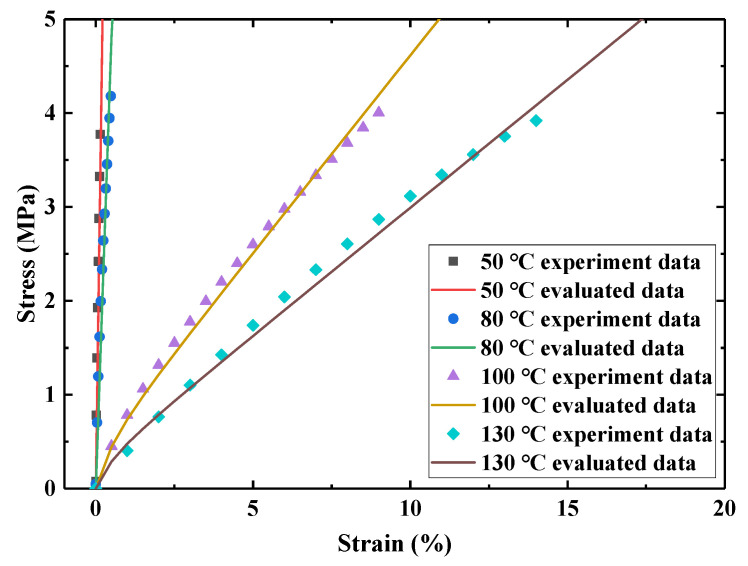
Evaluation curves of hyperelastic parameters at different temperatures.

In order to obtain the elastic parameters of E-SMP, the evaluation parameters of E-SMP at 50, 80, 100, and 130 °C were obtained using the ABAQUS material evaluation module. The evaluation results are shown in Figure 6 and the specific parameters are listed in Table 1. These parameters were used in the finite element model.

### 4.2. Viscosity Parameters Calibration

**Figure 7:**
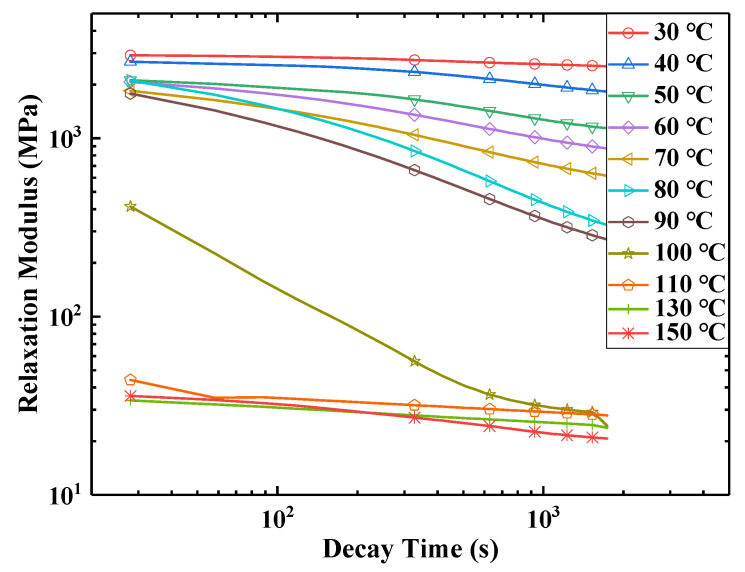
Elastic modulus versus time at different temperatures.

**Figure 8:**
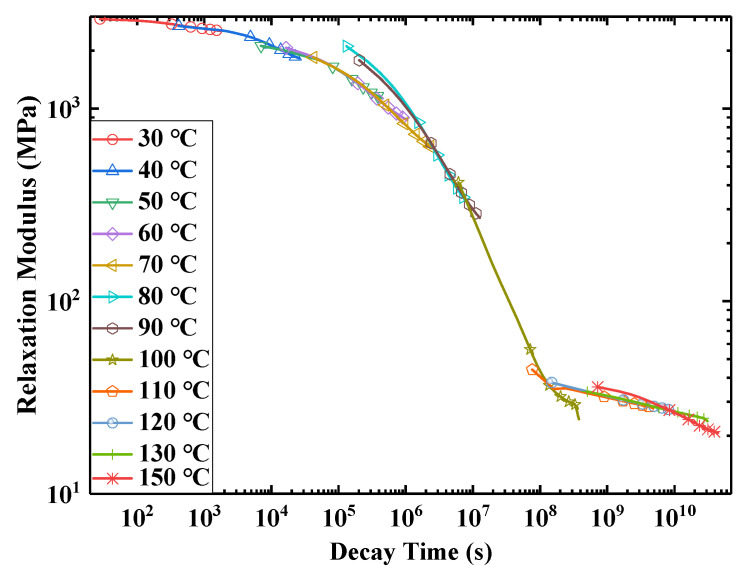
Translational change of the modulus at 30 °C.

**Figure 9:**
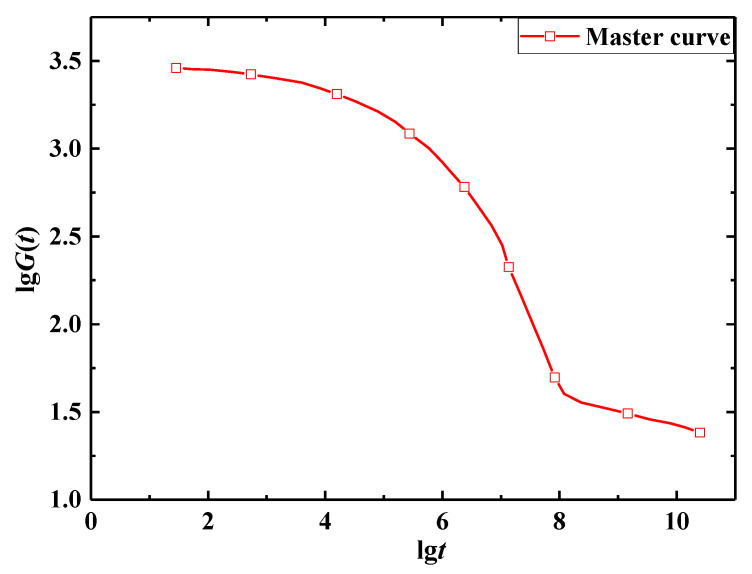
Master curve of the relaxation modulus at 30 °C.

**Figure 10:**
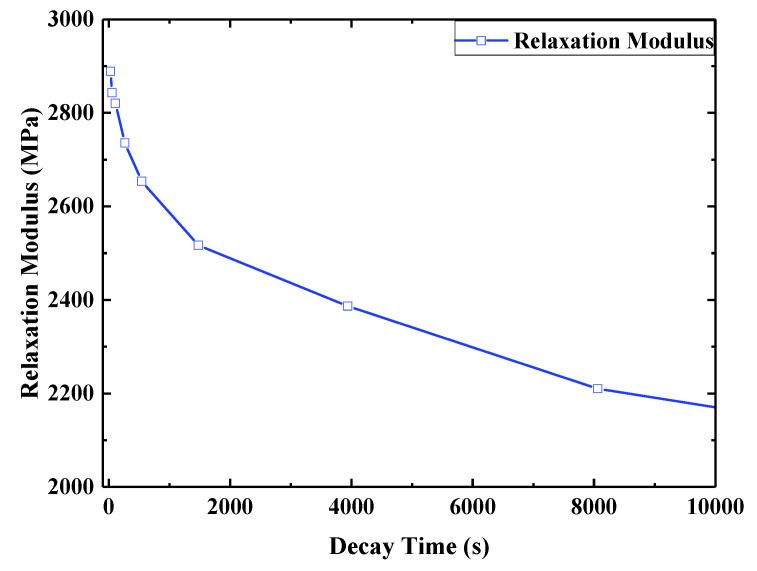
Relaxation modulus at the 30 °C.

According to the stress relaxation test data for E-SMP, by assuming that the volume of the E-SMP sample remains constant before and after the loading, the curves of each group of test data were obtained, then the logarithmic transformation of the horizontal axis and vertical axis was carried out, as shown in Figure 7. Based on the TTEP [65,68], the obtained values at various temperatures were converted into the values at 30 °C through time scale conversion, as shown in Figure 8. The following master curve of the relaxation modulus at a reference temperature of 30 °C was obtained, as depicted in Figure 9. The relaxation modulus curve for E-SMP at a reference temperature of 30 °C was obtained by processing the coordinate axis, as shown in Figure 10.

**Figure 11:**
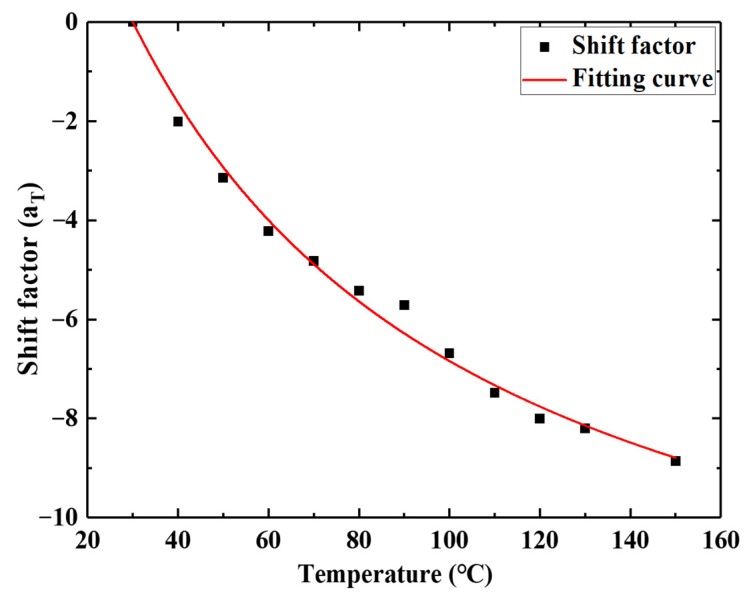
Migration coefficient fitting curve.

During the process of translation, according to the TTEP, we obtained the shift factor values of the relaxation at other temperatures when the reference temperature was 30 °C, as shown in Table 2. According to [49,65], the shift factor–temperature value can be expressed by the WLF equation. According to Equation (10), the parameters $C_{1}$ and $C_{2}$ can be obtained through curve fitting, which are shown in Figure 11. The parameters $C_{1}$ and $C_{2}$ were obtained as 14.664 and 77.636, as shown in Table 3, respectively.

**Figure 12:**
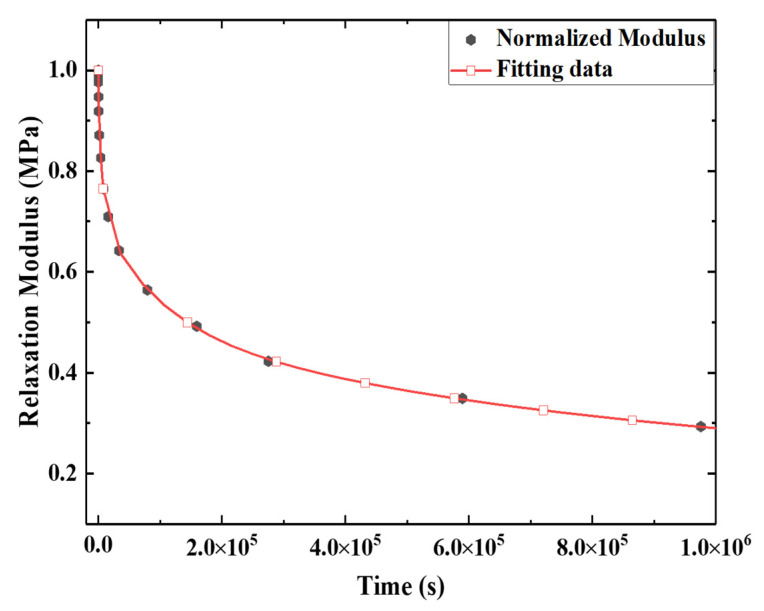
Fitting results for the master curve at 30 °C.

To obtain the viscoelastic parameters of E-SMP, according to Equation (6), the value of the master curve of the relaxation modulus at the reference temperature of 30 °C was fitted, as shown in Figure 11. For convenience of calculation, the relaxation modulus was normalized and $G_{\infty }$ was equal to 0.3 MPa. The modulus $G_{i}$ and relaxation time $\tau_{i}$ can be descried by the Prony series values of the E-SMP material, as shown in Table 4. The fitting curve of the Prony series value of the E-SMP material was compared with the relaxation modulus curve and the fitting effect was found to be feasible, as shown in Figure 12.

## 5. Verification of Numerical Analysis

**Figure 13:**
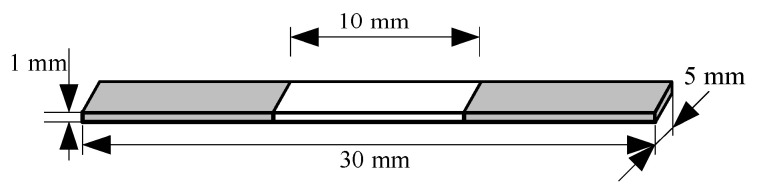
Schematic diagram of the test piece.

To verify the accuracy of the theoretical method, the three-dimensional model structure used for simulation was the same as that in the experiment. As shown in Figure 13, in order to match equipment range and for convenience of preparation, the model size was 30 mm × 5 mm × 1 mm. In the experiment, 10 mm sections at the two ends of the test piece were clamped and the middle 10 mm sections was tested in the actual experiments.

To maintain the same effect as that in the experiment, the two ends of the structure measuring 10 mm were constrained by MCP in the FEM. In the simulation, a 0.1 mm grid (C3D8RH; with an 8-node linear brick, hybrid and constant pressure, reduced integration, and hourglass control) was used. The software version was DS Simulia suite 2018, Multi.x64, run in Windows. Moreover, the specific material parameters were obtained from the above test data. The hyperelastic parameters are shown in Table 1, viscosity parameters in Table 4, WLF parameters in Table 3, and thermal expansion coefficients in Figure 4.

**Figure 14:**
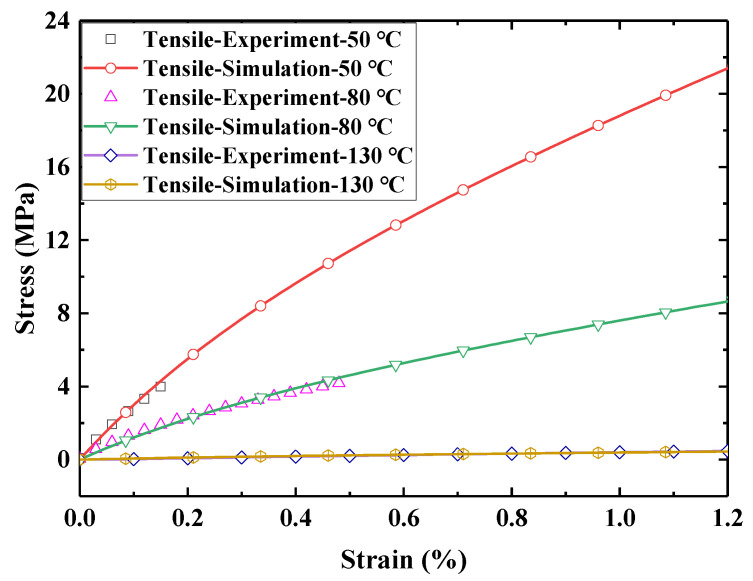
Comparison between uniaxial tension simulation and experimental data.

**Figure 15:**
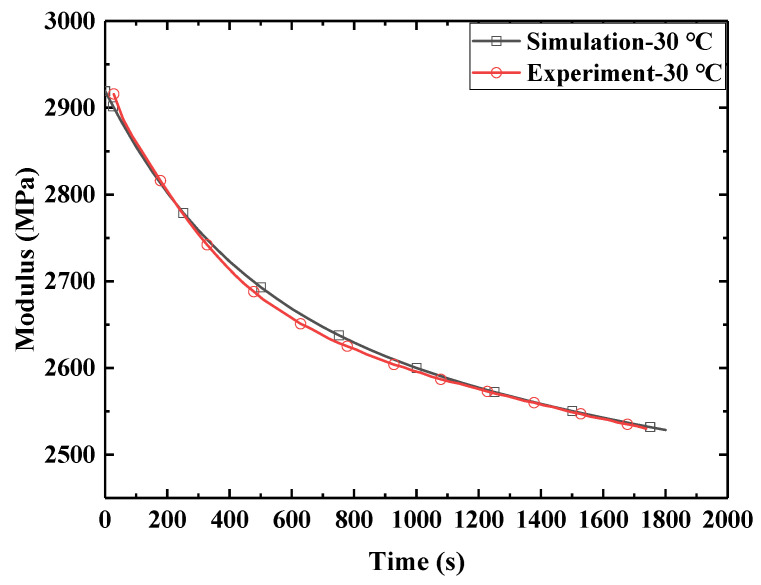
Comparison of stress relaxation with experimental data.

The simulation model was the same as that used in the actual test. A tensile rate of 15 mm/min was used in the tensile tests. The tensile simulation models at a low temperature of 30 °C, medium temperature of 80 °C, and high temperature of 130 °C were calculated and the results are shown in Figure 14. In the stress relaxation simulation at 30 °C, the loading rate was 2 mm/min, the tensile distance was 1 mm, and the strain was retained for 1800 s, the results for which are displayed in Figure 15. In Figure 14 and Figure 15, the simulation data are highly consistent with the experimental data; hence, the accuracy of the theoretical and simulation methods is demonstrated.

**Figure 16:**
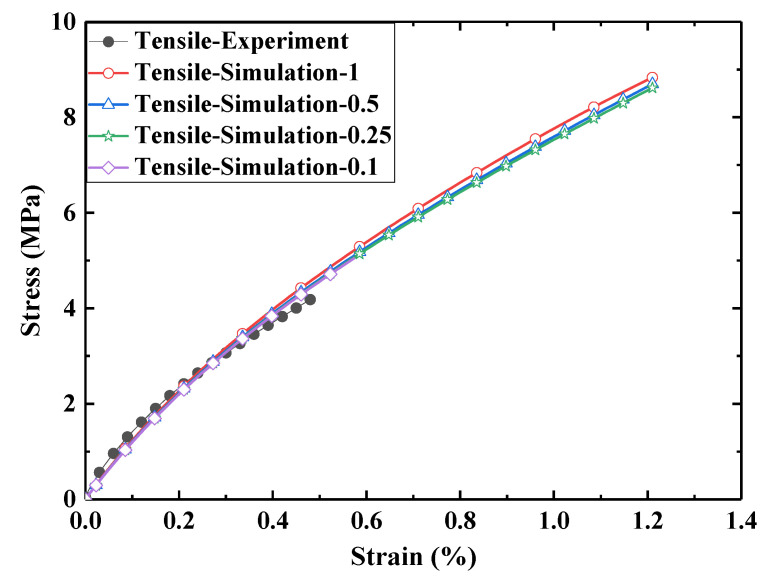
Verification of the grid independence.

Furthermore, the mesh independence was verified. Tensile test data at 50 °C were selected and grids with dimensions of 1 mm, 0.5 mm, 0.25 mm, and 0.1 mm were used. The results are presented in Figure 16. It can be seen from the figure that the smaller the grid, the closer the simulation results were to the experimental results; hence, to improve the computational efficiency, a 0.25 mm grid was selected.

## 6. Design and Analysis of Deployable Structure

### 6.1. Design and Analysis of the S-DS

**Figure 17:**
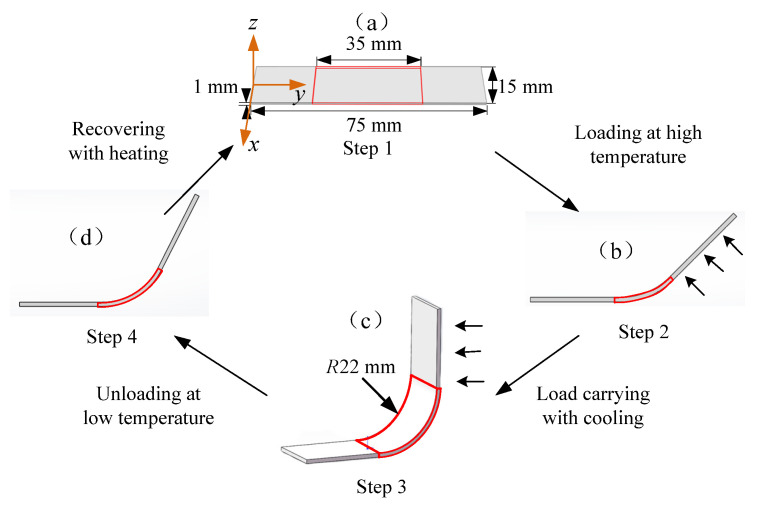
Shape memory flow chart for E-SMP: (**a**) the initial or permanent state; (**b**) the state of loading at high temperature; (**c**) the state in which one end is bent to 90°. The shape of S-DS recovers from state (**d**) to state (**a**) under thermal effect, when the S-DS samples are heated by electrification.

In space, lightweight and highly resilient driving components are extremely important for the deployment of large structures [18,19]. In this paper, E-SMP was used to design S-DS and F-DS. The structures not only possess a shape memory function, but also have a higher glass transition temperature (110 °C) and higher modulus (2350 MPa) at low temperatures. In addition, the E-SMP structure can be electrically driven and the deployment steps and process of the F-DS can be controlled by the loading voltages; therefore, the E-SMP structure material is suitable for the components of the space structure. The structure of the S-DS and the specific deformation process are illustrated as shown in Figure 17.

(1) Finite element analysis

The above calculation method and fitting parameters were used in the simulation model of the S-DS. In this simulation, the grid type was C3D8RH, with a size of 0.25 mm. The left end was fitted with a completely fixed restraint, while the right section was subjected to a 90° bending moment load during loading. The material parameters were the same as those used in the validation analysis and the thermal expansion effect was considered. According to the flow chart in Figure 17, the analysis was carried out in four steps, as follows:

(1) Loading at high temperature: At 150 °C, one end of the driving structure was fixed and the other end was loaded and bent to 90°, for which the analysis time was 100 s;
(2) Load carrying with cooling: Keeping the load unchanged when the temperature was reduced from 150 °C to 30 °C, the analysis time was 100 s;
(3) Unloading at low temperature: The temperature was kept at 30 °C, then the material was cooled for 1800 s and unloaded;
(4) Recovery with heating: There was no external load interference, the temperature increased from 30 °C to 150 °C, and the analysis time was 100 s.

(2) Analysis of results

**Figure 18:**
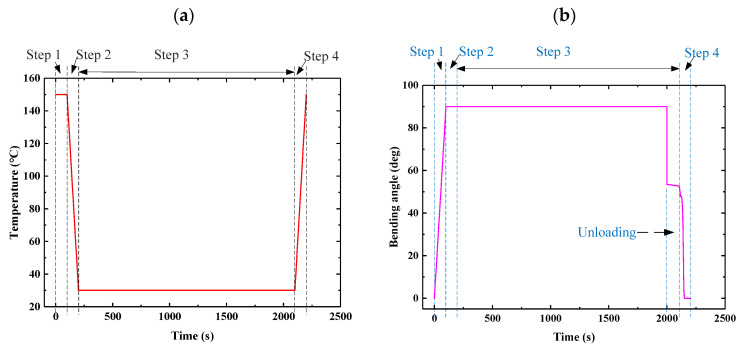
Response characteristic curves for E-SMP: (**a**) temperature–time curve; (**b**) bending angle–time curve.

The response characteristics of S-DS were obtained and the response curves of the temperature–time and bending angle–time relationships are shown in Figure 18. According to [55], the effect of the heating rate on the recovery of E-SMP can be ignored, further leading to the linear behavior of the temperature curves in Figure 18a. It can be seen from Figure 18b that in step 1, the structural material was rubberized at the temperature of the driving structure, which was maintained at 150 °C, while the structure bent to 90°. In step 2, the structure material was glassy when keeping the loading unchanged and the temperature was reduced to 30 °C. In step 3, the sample was kept in isothermal state at 30 °C for 1800 s in order to be cooled sufficiently, then the load was removed. Because of the high strength of the material at low temperatures, the structure recovered under a low-temperature elastic force when the load was removed. In step 4, the S-DS gradually reversed to the initial state under the thermal effect by increasing the temperature.

**Figure 19:**
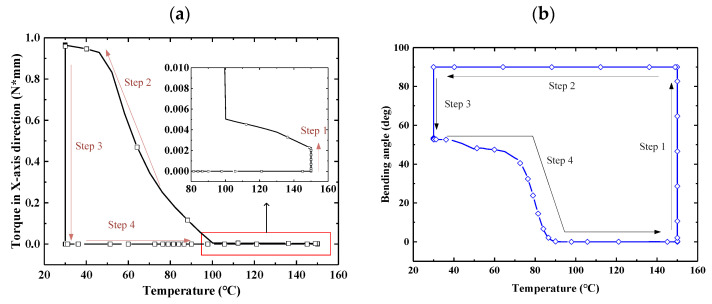
Shape memory characteristic diagram for E-SMP: (**a**) *X*-axis direction–temperature curves; (**b**) bending angle–temperature curve.

To describe the shape memory characteristics of the S-DS in a clear manner, the torque in the *X*-axis direction–temperature curve and bending angle–temperature curve for one end of the S-DS were plotted, as shown in Figure 19. The shape memory characteristics of the S-DS under the action of temperature can be clearly observed in the figure. As shown in Figure 19a, when the structure was loaded at a high temperature, the *X*-axis torque of the loading end was 0.05 N·mm. After cooling, the torque reached 0.98 N·mm. As shown in Figure 19b, the structure rebounded from 90° to 57° under a low-temperature elastic force when the load was removed. These results demonstrate that the shape retention rate of E-SMP needs to be improved.

**Figure 20:**
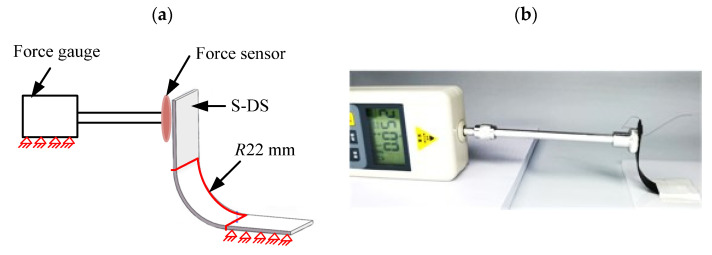
Recovery force measurements for S-DS: (**a**) schematic diagram of the recovery force measurement of S-DS; (**b**) experimental diagram of the recovery force measurement of S-DS.

The recovery force at one end of S-DS was measured when it was heated and recovered. The specific measurement method is shown in Figure 20. One end of the S-DS was fixed and the other end was loaded and bent to 90°. The bent end was measured by force sensor when S-DS was electrified and heated. The measured recovery forces are shown in Table 5. The average recovery forces was 0.049 N; however, the recovery force was 0.045 N in the simulation, while the *X*-axis torque of S-DS with bending radius of 22 mm was 0.98 N·mm, as shown in Figure 19a. Compared with the experimental results, the error of the numerical calculation of the recovery force at one end of S-DS was 8%. This also indirectly proved the feasibility of the numerical calculation method.

**Figure 21:**
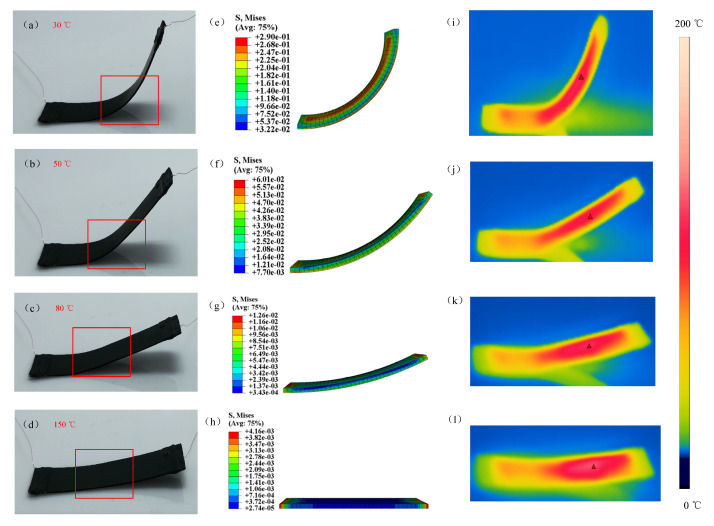
Simulation analysis chart and temperature field diagram for E-SMP during recovery: (**a**–**d**) deformation of the S-DS at 30, 50, 80, and 150 °C; (**e**–**h**) simulated stress cloud diagram of the S-DS at 30, 50, 80, and 150 °C; (**i**–**l**) experimental temperature test at 30, 50, 80, and 150 °C.

Furthermore, the simulation model for the S-DS was compared with the experimental verification. In this study, we mainly observed the deformation of the S-DS with the temperature increase (Figure 21a–d) and compared the simulated stress cloud diagram (Figure 21e–h) and the experimental temperature test (Figure 21i–l), which are shown in Figure 21. In Figure 21, the dynamic deformation of the S-DS at 30, 50, 80, and 150 °C can be visually observed. The main deformed parts of the actual structure (red box in the figure) were simulated and the results are depicted in Figure 21e–h. The simulated deformation was consistent with the actual structure, proving the effectiveness of the calculation method.

### 6.2. Design and Analysis of the F-DS

**Figure 22:**
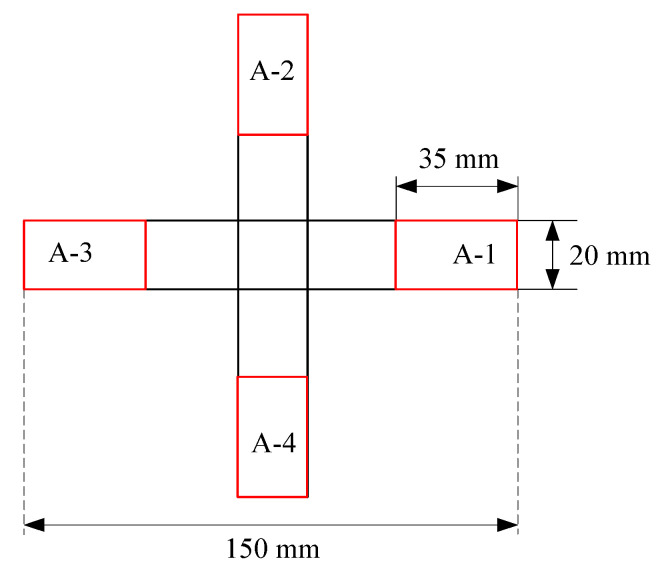
Schematic diagram of the F-DS.

The three-dimensional F-DS driving model of the E-SMP is shown in Figure 22, in which the thickness of the sample is 1 mm. The red frames in the figure represents the main driving component. In A-X, X is the number of single arms.

(1) Analysis of the simultaneous driving process

The calculation method and material parameters were consistent with those of the S-DS and the analysis steps of the F-DS were the same as the S-DS.

**Figure 23:**
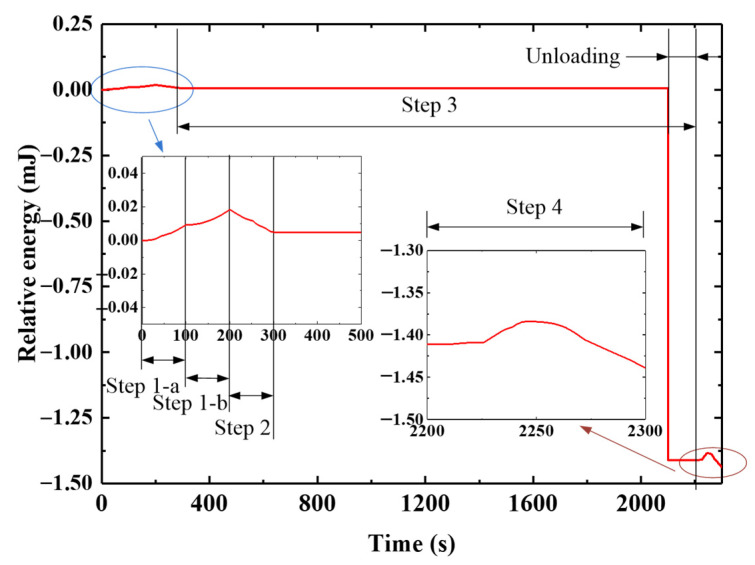
Internal response energy curve for the F-DS.

**Figure 24:**
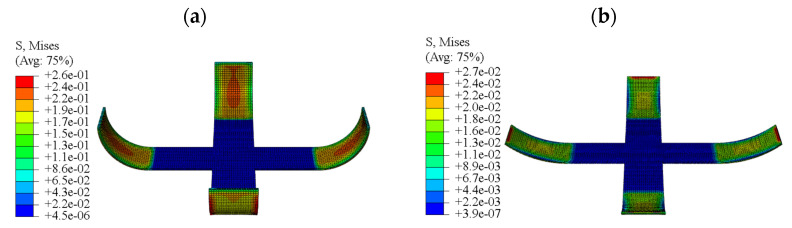
Response stress diagram for the F-DS: (**a**) stress cloud map of the F-DS subjected to external loads; (**b**) high-temperature recovery stress cloud map of the F-DS.

The F-DS was analyzed with ABAQUS based on the coupled FEM. Based on Equation (5), the relative internal energy curve of the F-DS in the response process was obtained, as shown in Figure 23. To more clearly express the changes in F-DS under temperature and external load, it was assumed that the internal energy of F-DS at normal atmospheric temperature was 0 mJ. The internal energy of the F-DS increased when the temperature increased or the external load was applied. In Figure 23, the ambient temperature was increased in step 1-a. In this process, the temperature was increased from 30 to 150 °C and the internal energy of the F-DS increased gradually. Step 1-b was the high-temperature loading stage. In this process, the internal energy of the F-DS increased gradually under the action of an external force. At this time, the force diagram of the F-DS was as shown in Figure 24a. Step 2 was the load carrying with cooling stage. In this process, the force of the F-DS remained unchanged and the temperature decreased from 150 to 30 °C. Owing to the change in the temperature field, the internal energy of the F-DS gradually decreased. Step 3 involved the cooling and unloading stages. The cooling was designed to relax the F-DS stress at a low temperature. After unloading, the internal energy of the F-DS decreased sharply. The response of the internal energy was obvious at low temperatures owing to the large modulus of the F-DS. The deformation was even easier at high temperatures, which was ascribed to the smaller modulus. To summarize, the F-DS released the external force load at high temperatures and the internal energy changed sharply at low temperatures in step 3. Step 4 was referred to as the recovery process with heating, in which the loaded temperature was increased from 30 to 150 °C. The high-temperature recovery stress cloud map of F-DS is shown in Figure 24. At this time, the four arms of the F-DS returned to their initial state under the action of thermal stress. Moreover, the internal energy tended to increase with the increase of temperature. The F-DS became rubber-like at high temperatures (>110 °C). The corresponding structure returned to its original shape and lost internal energy, meaning the internal energy curve tended to increase at the starting stage and then decrease in the step 4.

(2) Analysis of the step-by-step driving process

**Figure 25:**
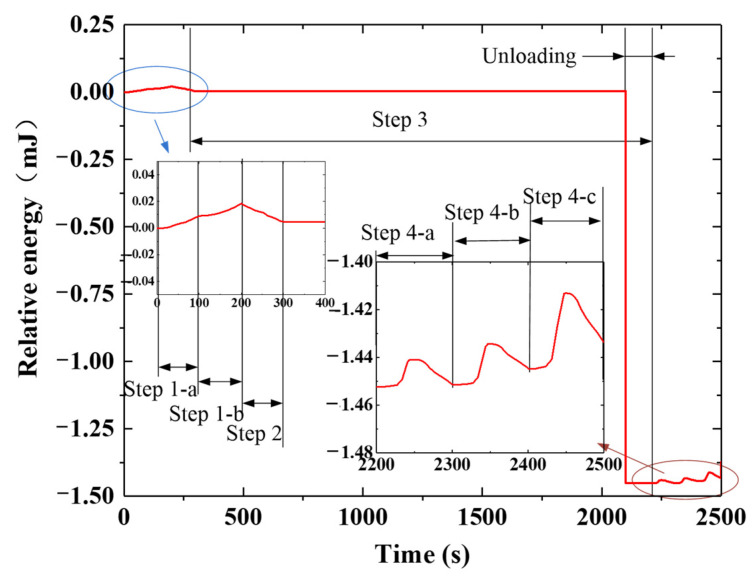
Response stress diagram of the F-DS with distributed drive.

**Figure 26:**
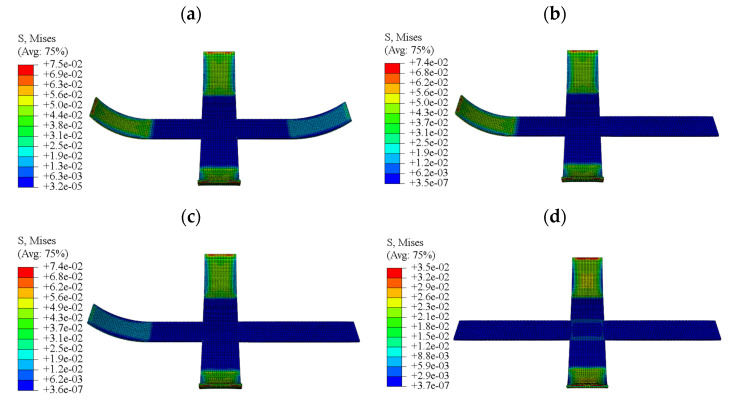
Response stress diagram of the F-DS with step recovery drive: (**a**) stress cloud map showing A-1 being driven; (**b**) stress cloud map showing A-1 being driven to completion; (**c**) stress cloud map showing A-3 being driven; (**d**) stress cloud map showing A-2 and A-4 being driven.

In this section, the step-by-step driving process of the F-DS was calculated and analyzed using the calculation method and material parameters that were used for the S-DS. The internal energy curve of the F-DS in the response process was obtained, as shown in Figure 25. In the figure, steps 1 to 3 were the same as the simultaneous driving of the F-DS; however, in step 4-a, A-1 was heated to characterize the recovery process. The stress diagram for A-1 at a certain time in the recovery process is shown in Figure 26a,b. At this time, the internal energy of the F-DS increased under the action of the thermal effect; however, the material became rubbery with the increase in temperature and internal energy was released to restore its original shape. The internal energy of the F-DS first increased and then decreased during this process. Similarly, step 4-b was the heating recovery process for A-3. The stress cloud in this process is shown in Figure 26c. Step 4-c was the process for heating A-2 and A-4 simultaneously. The stress cloud in this process is shown in Figure 26d. It can be clearly seen that the overall internal energy of the F-DS increased gradually with the step-by-step temperature loading.

## 7. Conclusions

Consequently, in this study we established a coupled FEM based on the generalized Maxwell model and TTEP. The thermodynamic behavior and shape memory effects of the S-DS and F-DS of the electrically driven epoxy resin SMPs prepared in the laboratory were studied and analyzed using this method. Through comparison of the experimental and simulation data of the tensile and S-DS recovery forces, the coupled FEM based on the generalized Maxwell model and TTEP was able to accurately and reliably describe the thermodynamic behavior and shape memory effect of the SMP structure, with an error of about 8%. The F-DS can be driven step-by-step under the same environment and temperature and can be used in large deployable structures in space satellites. This method can be used to provide novel ideas to facilitate future research on epoxy-shaped memory composites.

There are also some shortcomings in this paper. The effect of the heating rate on the recovery of the E-SMP was ignored and the shape recovery rate was low, which will be comprehensively considered in our future studies.

## Figures and Tables

**Table 1:** Evaluation of neo-Hookean parameters.

Temperature (°C)	C10
50	5.548
80	2.285
100	5.99 × 10^−4^
130	4.25 × 10^−4^

**Table 2:** Shift factor values at different temperatures with a reference temperature of 30 °C.

Temperature (°C)	Shift Factor	Temperature (°C)	Shift Factor
30	0	90	−5.71
40	−2.01	100	−6.68
50	−3.14	110	−7.48
60	−4.22	120	−8.01
70	−4.82	130	−8.20
80	−5.42	150	−8.86

**Table 3:** Parameters of the WLF equation.

Reference Temperature (°C)	$C_{1}$	$C_{2}$
30	14.664	77.636

**Table 4:** Prony series fitting values of E-SMP materials.

g_iProny	tau_iProny
9.89 × 10^−4^	436.82
0.191	9056.2
0.215	1.006 × 10^5^
0.295	8.416 × 10^5^

**Table 5:** Measurement data for the recovery forces.

Group	Recovery Force (N)
1	0.041
2	0.045
3	0.047
4	0.052
5	0.055
6	0.058
Average value	0.049

## Nomenclature

DMA	Dynamic mechanical analyzer
E-SMP	Shape memory polymer materials based on epoxy resin
FEM	Finite element method
F-DS	Four-arm deployment structure
SMPs	Shape memory polymers
S-DS	Single-arm deployment structure
SMPC	Shape memory polymer composite
TTEP	Time–temperature equivalence principle
UMAT	User-defined material subroutine
WLF	Williams–Lendel–Ferry equation
$T_{g}$	Glass transition temperature
